# Galanin and Neuropeptide Y Interaction Enhances Proliferation of Granule Precursor Cells and Expression of Neuroprotective Factors in the Rat Hippocampus with Consequent Augmented Spatial Memory

**DOI:** 10.3390/biomedicines10061297

**Published:** 2022-06-01

**Authors:** Marina Mirchandani-Duque, Miguel A. Barbancho, Alexander López-Salas, Jose Erik Alvarez-Contino, Natalia García-Casares, Kjell Fuxe, Dasiel O. Borroto-Escuela, Manuel Narváez

**Affiliations:** 1 Instituto de Investigación Biomédica de Málaga, Facultad de Medicina, Universidad de Málaga, 29071 Malaga, Spain; marina.md97@uma.es (M.M.-D.); mabarbancho@uma.es (M.A.B.); 0611059553@uma.es (A.L.-S.); 0611059544@uma.es (J.E.A.-C.); nagcasares@uma.es (N.G.-C.); 2 Department of Neuroscience, Karolinska Institute, 17177 Stockholm, Sweden; kjell.fuxe@ki.se; 3 Department of Biomolecular Science, Section of Physiology, University of Urbino, 61029 Urbino, Italy

**Keywords:** neuropeptide Y1 receptor, galanin 2 receptor, receptor–receptor interaction, neurogenesis, dorsal hippocampus, neuroprotection, BDNF, Bcl-2, neurodegenerative diseases, spatial memory

## Abstract

Dysregulation of hippocampal neurogenesis is linked to several neurodegenereative diseases, where boosting hippocampal neurogenesis in these patients emerges as a potential therapeutic approach. Accumulating evidence for a neuropeptide Y (NPY) and galanin (GAL) interaction was shown in various limbic system regions at molecular-, cellular-, and behavioral-specific levels. The purpose of the current work was to evaluate the role of the NPY and GAL interaction in the neurogenic actions on the dorsal hippocampus. We studied the Y1R agonist and GAL effects on: hippocampal cell proliferation through the proliferating cell nuclear antigen (PCNA), the expression of neuroprotective and anti-apoptotic factors, and the survival of neurons and neurite outgrowth on hippocampal neuronal cells. The functional outcome was evaluated in the object-in-place task. We demonstrated that the Y1R agonist and GAL promote cell proliferation and the induction of neuroprotective factors. These effects were mediated by the interaction of NPYY1 (Y1R) and GAL2 (GALR2) receptors, which mediate the increased survival and neurites’ outgrowth observed on neuronal hippocampal cells. These cellular effects are linked to the improved spatial-memory effects after the Y1R agonist and GAL co-injection at 24 h in the object-in-place task. Our results suggest the development of heterobivalent agonist pharmacophores, targeting Y1R–GALR2 heterocomplexes, therefore acting on the neuronal precursor cells of the DG in the dorsal hippocampus for the novel therapy of neurodegenerative cognitive-affecting diseases.

## 1. Introduction

Differing from the ancient notion that the number of neurons in the brain remains fixed after prenatal and neonatal development, new neurons can be generated in the adult brain via a process known as neurogenesis. Neurogenesis is one mechanism of neuronal plasticity, the ability of the nervous system to reorganize its structure, function, and connections in response to extrinsic or intrinsic stimuli [1]. Among the neurogenic areas mapped in the human brain, hippocampal formation plays a significant role in brain function [2]. This neurogenic niche is located in the hippocampus’ dentate gyrus (DG), and accumulating evidence demonstrates its relevance in preserving many physiological functions during adulthood. Moreover, the hippocampus shows a functional separation, where a more anterior portion (ventral, in rodents) is related to stress and modulation of emotional behavior, and the posterior part (dorsal, in rodents) is linked to cognitive functions and participates in declarative memory, spatial navigation, and contextual learning [3,4,5]. In fact, adult human neurogenesis is preserved throughout human life, even until the ninth decade of life, being necessary to distinguish physiological from pathological aging and the associated cognitive impairment [6,7,8]. However, aging is a significant risk for the development of cognitive decline and neurodegenerative disorders since the homeostasis of the neurogenic niche is altered. Thus, dysregulation of hippocampal neurogenesis is linked to several brain disorders, such as age-dependent cognitive decline, Alzheimer’s disease (AD), amyotrophic lateral sclerosis, Huntington’s disease, Parkinson’s disease, dementia with Lewy bodies, and frontotemporal dementia [6,8,9,10]. AD, the most frequent among neurodegenerative diseases, accounts for about 70% of dementia cases worldwide, that is, about 35 million people. Currently, no pharmacological treatment is available to cure or even significantly slow down the course of neurodegenerative diseases. Accordingly, boosting hippocampal neurogenesis in these patients emerges as a potential therapeutic approach to counteract the progression of these disorders [11].

Cell proliferation, neuronal differentiation, and survival are critical components of adult neurogenesis, tightly modulated by multiple intrinsic or extrinsic epigenetic factors that could promote or suppress neurogenesis. These factors can actively upregulate or downregulate the formation of new neurons during adulthood, conferring a pivotal role in understanding the importance of adult neurogenesis on physiological and pathological conditions [12]. However, physiologically occurring programmed cell death represents more than half of the differentiating neurons in the adult brain, where a combination of factors can serve to rescue or influence the neurogenic process. In this way, the neurogenic-promoting effects of neurotransmitters/neuropeptides (i.e., neuropeptide Y) and neurotrophic factors (i.e., BDNF) are ultimately mediated by interference with apoptosis-inducing signaling pathways (i.e., Bcl-2 family members) [13].

Neuropeptides are emerging as crucial regulators of neurogenic niche activities in health and disease. Among them, neuropeptide Y (NPY) is one of the most abundant neuropeptides in the nervous system. NPY is a 36-amino acid polypeptide highly conserved during phylogenesis and implicated in regulations of essential biological and pathophysiological functions, such as blood pressure, neuroendocrine secretions, feeding behavior, circadian rhythms, seizures, neuronal excitability, neuroplasticity, and memory [14]. Regarding hippocampal neurogenesis, a pro-neurogenic role of NPY on hippocampal stem cells has been evidenced both in vitro and in vivo [15,16]. Interestingly, an upregulated NPY mRNA expression level in the hippocampal dentate gyrus was shown after spatial learning tasks in rats [17]. In contrast, in aged rats, the decreased NPY expression in the hippocampus was followed by memory impairment and deterioration of neurogenesis [18]. Similarly, in AD patients, NPY receptor densities were reduced in hippocampal and cortical regions [19] and NPY levels in cerebrospinal fluid and plasma samples [20]. In this respect, the NPY Y1 receptors (Y1R) have been proposed as a critical target in enhancing dentate neurogenesis and spatial learning [21].

Moreover, in the trimethyltin (TMT)-induced model of hippocampal neurodegeneration, the brain-derived neurotrophic factor (BDNF) was significantly increased 24 h following treatment with NPY [22]. BDNF is a key molecule involved in neuroplasticity changes related to learning and memory. Moreover, BDNF appears to be crucial or, in some cases, even essential to mediate the neuroprotective effects of the above-mentioned neurodegenerative diseases by regulating different neurogenic processes [11,23,24]. Interestingly, BDNF can directly stimulate the expression of anti-apoptotic Bcl-2 protein, thereby leading to an increase in neurogenesis [13].

Accumulating evidence for NPY and galanin (GAL) interactions was shown in various limbic system regions at molecular-, cellular-, and behavioral-specific levels [25,26,27]. Like NPY, GAL is widely distributed in the central nervous system, where it has a variety of physiological effects [28]. Its effects on hippocampal neurogenesis were not elucidated until the demonstration that the GAL 2/3 receptor agonist, GAL 2–11, was proliferative and trophic on progenitor cells in vitro [29]. Concerning memory, depending on the dose or administration site, improvement in learning, lack of outcome, or even an inhibitory effect were observed after GAL administration [30]. Furthermore, it has also been shown that the GALR2 receptors mediate memory-improving and hippocampal toxicity-inhibiting effects in a rat model of AD [31]. Recently, we have described a facilitatory interaction between NPY and GAL through the formation of GALR2/Y1R heteroreceptor complexes. Moreover, GALR2 enhanced Y1R agonist-mediated antidepressant-like activities in the forced swimming test related to increased cell proliferation in the dentate gyrus of the ventral hippocampus [32].

The purpose of the current work was to evaluate the role of the NPY and GAL interaction in the neurogenic actions on the dorsal hippocampus. We studied the hippocampal cell proliferation effects mediated by GAL and Y1R agonists through the proliferating cell nuclear antigen (PCNA). To analyze the associated cellular mechanism, we assessed the expression of neuroprotective factors, BDNF, or the anti-apoptotic factor Bcl-2 on the dorsal hippocampal dentate gyrus (DG). Moreover, we studied the survival of neurons in a viability assay and analyzed the neurite outgrowth on hippocampal neuronal cells. Finally, the functional outcome of the NPY and GAL interaction on the dorsal hippocampus was evaluated in the object-in-place task, specific for spatial hippocampal memory.

## 2. Materials and Methods

### 2.1. Animals

Male Sprague-Dawley rats were purchased from CRIFFA (Barcelona; 200–250 g, 6–8 weeks). Animals had free access to food and water and were maintained under the usual 12 h dark/light cycle, with controlled temperature (22 ± 1 °C) and relative humidity (57–60%). Procedures for preclinical experiments were followed according to EU Directive 2010/63/EU and Spanish Directive (Real Decretory 53/2013) consents. All methods involving experimental treatments, housing, and maintenance of the animals were authorized by the Local Animal Ethics, Use, and Care Committee for the University of Málaga, Spain (2018-0010, 8 May 2018).

### 2.2. Drugs Used

Diluted peptides were recently prepared in artificial cerebrospinal fluid (aCSF, composition is (in mM): 120 NaCl, 20 NaH_2_CO_3_, 2 KCl, 0.5 KH_2_PO_4_, 1.2 CaCl_2_, 1.8 MgCl_2_, 0.5 Na_2_SO_4_, and 5.8 D-glucose, pH 7.4). aCSF as a vehicle has been used for control preparations. Galanin (GAL), the GALR2 agonist M1145, the Y1R receptor agonist [Leu^31^, Pro^34^]NPY, and the GALR2 antagonist M871 were acquired from Tocris Bioscience (Bristol, UK). A detailed report is accessible in the Supplementary Material on intracerebroventricular (icv) administration of peptides [25,26,27,32,33,34].

### 2.3. Evaluation of Hippocampal Cell Proliferation

Animals were distributed randomly into five experimental groups: (1) aCSF: control group, (2) GAL-treated group (3 nmol), (3) Y1R agonist-treated group receiving an NPYY1R agonist [Leu^31^,Pro^34^]NPY (3 nmol), (4) GAL+Y1R: group administered with both substances, and (5) GAL+Y1R+M871: group injected with GAL, [Leu^31^,Pro^34^]NPY, and the GALR2 antagonist (M871, 3 nmol) (N = 4 in each group). The doses indicated above are based on previously published protocols [25,26,27,32].

Twenty-four hours after the icv injection, rats were deeply anesthetized with pentobarbital (Mebumal, 100 mg/kg, i.p.) and transcardially perfused with 4% PFA (paraformaldehyde (wt./vol, Sigma Aldrich, St. Louis, MI, USA)). Through the dorsal hippocampus (posterior in primates) (from −1.60 to −5.30 Bregma; Paxinos and Watson, 2006 [33]), the brains were coronally sliced (30 μm-thick) using a Cryostat (HM550, Microm International, Walldorf, Germany).

The brain slices were treated for antigenical retrieval at 65 °C during 90 min in saline sodium citrate buffer (pH 6; 10 nM sodium citrate). After this procedure to remove endogenous peroxidases, the slices were treated 30 min in 0.6% H_2_O_2_. Then, slices were incubated at 4 °C overnight with a primary antibody against PCNA (1:1500, Sigma) in 2.5% donkey serum. After several washes with PBS, the slices were incubated with a secondary antibody for 90 min (biotinylated anti-mouse IgG, 1/200, Vector Laboratories). Then, ExtrAvidin peroxidase (1:100, Sigma, St. Louis, MO, USA) was used to amplify the specific signal for one hour at room temperature in darkness. Detection was performed with 0.05% diaminobenzidine (DAB; Sigma) and 0.03% H_2_O_2_ in PBS. After several washes, slices were mounted on gelatin-coated slides, dehydrated in graded alcohols, and cover-slipped with DePeX mounting medium (VWR, Radnor, PA, USA). PCNA-labeled cells were studied using the optical fractionator method in unbiased stereological microscopy (Olympus BX51 Microscope, Olympus, Denmark), as previously described [27] (see Supplementary Materials for details).

### 2.4. Assessment of Neuroprotective Factors

To study brain-derived neurotrophic factor- (BDNF) or the anti-apoptotic factor Bcl-2-immunoreactive (IR) cells, immunofluorescence was performed as follows). Rats were randomly allocated in different groups: (1) aCSF: control group, (2) GAL group, injected with galanin (3 nmol), (3) Y1R agonist group, administered with the Y1R agonist [Leu^31^,Pro^34^]NPY (3 nmol), (4) GAL+Y1R group, co-injected with both substances, and (5) GAL+Y1+M871 group, treated with GAL, the Y1R agonist, and the GALR2 antagonist (M871, 3 nmol) (n = 4 in each group). Twenty-four hours after icv injections, animals were perfused, and brain slices were processed as described above. An initial incubation with blocking (5% goat serum) and permeabilization (0.3% triton X100 in PBS) solutions was performed for 60 min each. Primary antibodies mouse anti-BDNF (Abcam, ab205067, 1:500) or mouse anti-Bcl-2 (Santa Cruz Biotechnology INC, Dallas, TX, USA, sc-7382, 1:200) were used to incubate the sections for 24 h, at 4 °C. Then, incubations were performed with proper secondary antibodies: rabbit anti-mouse DyLight 549 (Jackson inmunoResearch Laboratories, West Grove, PA, USA, 1:100) against anti-BDNF, or rabbit anti-mouse DyLight 488 (Jackson Laboratories InmunoResearch, 1:100) against anti-Bcl-2. Nuclei were detected with 4′,6-diamidino-2-phenylindole (DAPI) (1 µg/mL). Sections were mounted on slides with a fluorescent mounting medium (Dako). Quantitative analyses of the BDNF- and Bcl-2-immunostained cells in the dentate gyrus of the dorsal hippocampus were performed as described in [35].

### 2.5. Hippocampal Neuronal Cells’ Viability Analysis

The MTS assay determined cell viability using the CellTiter 96 Aqueous One Solution Cell Proliferation Assay kit (G3580, Promega, Madison, WI, USA). This assay determines the levels of cellular 3-[4,5-dimethylthiazol-2-yl-5-(3-carboxymethoxyphenyl)-2-(4-sulfo- phenyl)-2H-tetrazolium, inner salt] (MTS) reduction to formazan, a measure of mitochondrial function. Cultured hippocampal neurons (7 days in vitro) were collected, and approximately 2 × 10^4^ cells per well were plated into 96-well plates, which were treated for 0, 8, 16, and 24 h, respectively, as described below, in B27-deprived medium, and then 20 µL of MTS solution was added to each well. Plates were incubated at 37 °C for 3 h. The absorbance was determined with a POLARstar Optima plate reader (BMG Labtech) at 490 nm, directly proportional to the number of living cells in the culture. This protocol was performed as described in [36]. Detailed descriptions are available in the Supplementary Materials on cell hippocampal culture and reagents’ pharmacologic conditions.

### 2.6. Analysis of Neurite Outgrowth

A different set of cultured hippocampal neurons was grown and treated for 24 h under specific pharmacologic conditions. Treated hippocampal cells were divided into experimental groups: (1) Control group, (2) M1145-treated group (100 nM), (3) Y1R agonist-treated group receiving an NPYY1R agonist [Leu^31^,Pro^34^]NPY (100 nM), (4) GAL+Y1R: group administered with both substances, and (5) GAL+Y1+M871: group injected with GAL, [Leu^31^,Pro^34^]NPY, and the GALR2 antagonist (M871, 1 µM). Hippocampal neurons were stained with Neuro-Chrom Pan Neuronal Marker (ABN2300, 1:100, Sigma-Aldrich; Merck Life Science S.L.U., Darmstadt, Germany), and cell nuclei were counterstained with DAPI (blue). Acquisition of microscopy images and morphometric quantifications was performed as previously described in [37].

### 2.7. Assessment of Spatial Memory in Rats

To evaluate spatial hippocampal memory, the object-in-place task was developed based on spontaneous object exploration behaviors [38]. Since rodents are more physiologically adapted to dry land tasks, the object-in-place task may better reveal learning/navigation behaviors than the Morris water maze task. The Morris water maze task is more stressful than the object-in-place task maze, which can disturb learning and memory performance [39]. Rats were exposed to the task to assess memory consolidation at 24 h using a plastic open field, 100 × 100 × 60 cm (length × width × height), under dim light. Rats were single-housed during the behavioral period. The task trials contain three phases: habituation, training, and test [40,41] as follows:

Habituation: animals were handled for two days, then familiarized with the empty arena for 10 min (1 trial, 10 min).

Training: Every animal was placed in the middle of the arena 24 h after the habituation. The rats were allowed to explore four distinct objects which could not be displaced, during 3 min. The objects were placed in the corners of the arena 10 cm from the sidewall and were different in color and shape, with a similar weight and size. The objects were cleaned with 5% ethanol after the trial.

Test: Two objects were exchanged 24 h post-training and then the animals were re-exposed to explore the objects (1 trial, 3 min). The time spent sniffing or touching the object with the nose or forepaws was described as exploration. The time spent exploring the objects in the exchanged location (C) compared with the time spent exploring the objects in the same place (S) represented the discrimination ability. Then, the discrimination ratio was calculated as DI = (C − S)/(C + S). Intact object-in-place memory occurs when the animal spends more time examining the two objects in different locations than the same ones. The animals’ behavior was scored and analyzed blind to the treatment, using an overhead video camera and the Raton Time 1.0 software (Fixma S.L., Valencia, Spain). The video-tracking software EthovisionXT (Noldus, Wageningen, Nederland) was used to analyze the locomotor activity. Objects’ locations were counterbalanced between trials and between rats. Furthermore, the arena and the objects were carefully cleaned with 5% ethanol between sessions. The treatments were injected 24 h before the test phase. Moreover, the total exploration time (Supplementary Table S1) and the locomotor activity (Supplementary Table S2) between the animal groups were analyzed to validate that the treatments did not affect the exploration ability of the rats.

### 2.8. Statistical Analysis

The data obtained are showed as mean ± SEM, and sample number (n) is detailed in figure legends. GraphPad PRISM 8.0 (GraphPad Software, La Jolla, CA, USA) was used to analyze all data. One-way analysis of variance (ANOVA) followed by the Newman–Keuls comparison post-test was performed when indicated in figure legends. For comparing two experimental conditions, Student’s unpaired *t*-test statistical analysis was achieved on the hippocampal survival analysis. Paired Student’s *t*-tests (two-tailed) were used to study the discrimination ability between the objects of the animals in the object-in-place task. Differences were considered significant at *p* < 0.05 (* *p* < 0.05, ** *p* < 0.01, *** *p* < 0.001).

## 3. Results

### 3.1. GAL and Y1R agonist Co-Administration Increased Cell Proliferation in the Dorsal Hippocampus

We assessed the impact of GAL and Y1R agonist co-injection on adult dorsal hippocampal cell proliferation by using the proliferating cell nuclear antigen (PCNA).

**Figure 1:**
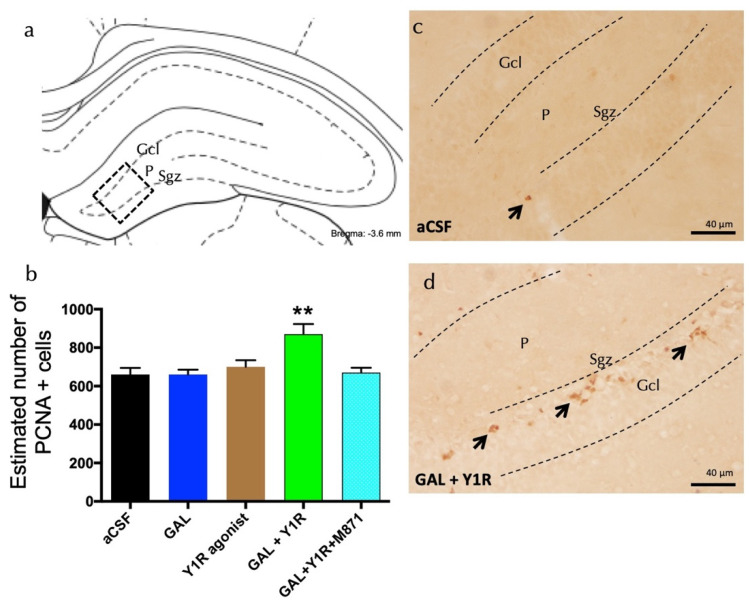
Co-administration of galanin and Y1R agonists increases cell proliferation in the dentate gyrus of adult rats. Proliferating cell nuclear antigen immunolabeling (PCNA+) in the dentate gyrus of the dorsal hippocampus, after the intracerebroventricular (icv) administration of galanin (GAL) and Y1R receptor agonists, either alone or in combination with or without the GAL 2 receptor antagonist (M871). (**a**,**d**) The majority of the PCNA-IR cells were located in the sub-granular zone (Sgz) of the dentate gyrus at the border between the granular cell layer (Gcl) and the polymorphic layer (P) of the dentate gyrus in the dorsal hippocampus. They appeared as groups of 3–4 cells (Bregma: −3.6 mm, according to the Paxinos and Watson stereotaxic atlas [33]). (**b**) Quantification of total PCNA-IR cells in the dorsal hippocampal dentate gyrus. Data represent mean ± SEM, showing the differences between groups after the injections of aCSF, GAL, the Y1R agonist [Leu^31^,Pro^34^]NPY, or the co-administration of both substances with or without M871. GAL and the Y1R agonist co-administration augmented the number of PCNA+ cells in the dorsal hippocampus compared to the lack of effects of the two peptides alone and the aCSF group. Additionally, this effect was blocked by the GALR2 antagonist M871. ** *p* < 0.01 vs. the rest of the groups according to one-way ANOVA followed by Newman–Keuls post-hoc test. N = 4 in each group. Statistical values are presented in Supplementary Table S1. GAL and Y1R agonist co-injection (**d**) increased the PCNA-IR cells in Sgz in the dentate gyrus compared with the control group (**c**). Arrows indicate examples of clusters of PCNA+ nerve cells. Dashed lines outline the Gcl of the dentate gyrus. Abbreviations: aCSF = Control (artificial cerebrospinal fluid), GAL = galanin (3 nmol), Y1R agonist = Y1R receptor agonist [Leu^31^, Pro^34^]NPY (3 nmol), GAL + Y1R = co-administration of GAL and Y1R, GAL + Y1R + M871 = co-administration of GAL, Y1R, and GALR2 antagonist M871 (3 nmol).

GAL and Y1R agonist co-injection significantly increased cell proliferation, as evidenced by the number of PCNA-IR profiles, specifically in the sub-granular zone (Sgz) of the dentate gyrus compared with GAL, Y1R agonist, and the aCSF control groups (one-way ANOVA, F4, 15 = 6.24, *p* < 0.01, Newman–Keuls post-hoc test: *p* < 0.01) (Figure 1a,b,d). The addition of GALR2 antagonist M871 completely blocked the GAL and Y1R agonists’ action in the dentate gyrus (Newman–Keuls post-hoc test: *p* < 0.01) (Figure 1b), indicating the participation of GALR2 in the GAL/NPYY1R agonist interaction to stimulate cell proliferation on the dorsal hippocampus.

However, the icv injection of the Y1R agonist induced no significant changes in the number of PCNA-IR profiles in the Sgz of the dentate gyrus (Figure 1a,b) compared with the control group. The administration of GAL alone lacked effects on the numbers of PCNA-IR profiles (Figure 1b) compared with the control group (Figure 1a–c). Statistical values are presented in Supplementary Table S1.

### 3.2. Enhanced Cell Proliferation Is Related to Increased Neuroprotective Factors upon GAL and Y1R agonist Coactivation

To analyze the cellular mechanism related to the observed effects on cell proliferation, we study neuroprotection mediated by BDNF or Bcl-2 expression on the dorsal hippocampal dentate gyrus (DG) after GAL and/or NPYY1R agonist administration.

**Figure 2:**
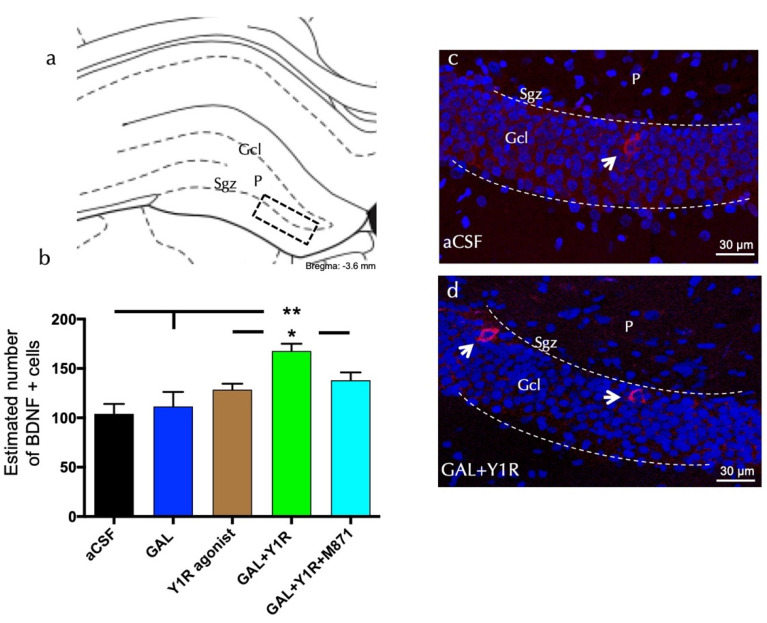
Galanin and the Y1R agonist effects on hippocampal brain-derived neurotrophic factor-immunoreactive (BDNF-IR) cells of the dentate gyrus (DG) hippocampal region. (**a**) BDNF-IR cells were located mainly in the sub-granular zone (Sgz) of the dentate gyrus at the border of the granular cell layer (Gcl), and some scattered cells were found in the polymorphic layer (P) of the dentate gyrus in the dorsal hippocampus (Bregma: −3.6 mm, according to the Paxinos and Watson [33] stereotaxic atlas). (**b**) Quantitative morphometric analysis of BDNF-IR cells of the DG. GAL and Y1R agonist icv co-administration significantly increased BDNF-IR cells in the dorsal DG. This effect was counteracted in the presence of the GALR2 antagonist M871. * *p* < 0.05 vs. Y1R agonist and GAL + Y1R + M871; ** *p* < 0.01 vs. aCSF and GAL according to one-way ANOVA followed by Newman–Keuls post-hoc test. The vertical lines from the horizontal line above the bars indicate the inter-group comparisons. Data are expressed as mean ± SEM, n = 4. Statistical values are presented in Supplementary Table S1. (**c**,**d**) Representative microphotographs of the significant increment in the BDNF-positive cells in the DG after GAL and Y1R agonist co-injection (**d**) compared with the control group (**c**). The cells in red are BDNF-positive using confocal laser microscopy. White arrows point to BDNF-IR cells. Dashed lines outline the Gcl of the dentate gyrus. The nuclei are shown in blue by DAPI. Abbreviations: aCSF = Control (artificial cerebrospinal fluid), GAL = galanin (3 nmol), Y1R agonist = Y1R receptor agonist [Leu^31^, Pro^34^]NPY (3 nmol), GAL + Y1R = co-administration of GAL and Y1R, GAL + Y1R + M871 = co-administration of GAL, Y1R, and GALR2 antagonist M871 (3 nmol).

BDNF-positive cells were found specifically in the sub-granular zone (Sgz) of the dorsal hippocampus, and some scattered cells were observed in the polymorphic layer (P) of the dorsal DG (Figure 2a). Quantification of BDNF cells demonstrated an increase in the density of the BDNF-positive cells after the co-injection of GAL and YR1 agonists compared to control (one-way ANOVA, F4, 15 = 6.59, *p* < 0.01, Newman–Keuls post-hoc test: *p* < 0.01), GAL (Newman–Keuls post-hoc test: *p* < 0.01), or the YR1 agonist alone (Newman–Keuls post-hoc test: *p* < 0.05) (Figure 2a–d). The injection of GAL or the Y1 agonist alone lacked effects on the number of BDNF-positive cells in the dorsal DG. Similar to the PCNA-IR profile’s response described above, the presence of the GALR2 antagonist M871 completely blocked this increase (Newman–Keuls post-hoc test: *p* < 0.05) (Figure 2b), demonstrating the involvement of GALR2 in this interaction. Statistical values are presented in Supplementary Table S1.

**Figure 3:**
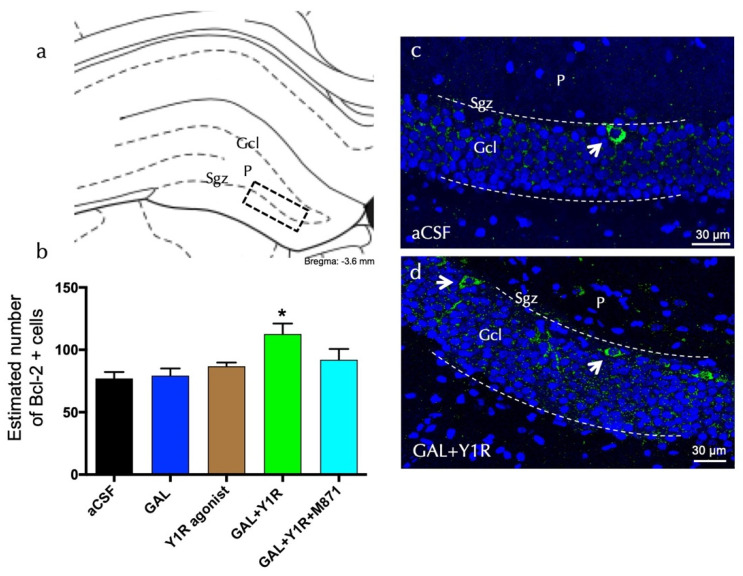
Dentate gyrus (DG) hippocampal anti-apoptotic Bcl-2-immunoreactive cells (Bcl-2-IR) after galanin and the Y1R agonist co-administration. (**a**) Bcl-2-IR cells were located in the sub-granular zone (Sgz) of the dentate gyrus at the border between the granular cell layer (Gcl) and the polymorphic layer (P) of the dentate gyrus in the dorsal hippocampus (Bregma: −3.6 mm, according to the Paxinos and Watson [33] stereotaxic atlas). (**b**) Quantitative morphometric analysis of Bcl-2-IR cells of the DG. GAL and Y1R agonist icv co-administration significantly increased Bcl-2-IR cells in the dorsal DG. This effect was blocked by treatment with the GALR2 antagonist M871. * *p* < 0.05 vs. the rest of the groups according to one-way ANOVA followed by Newman–Keuls post-hoc test. Data are expressed as mean ± SEM, n = 4. Statistical values are presented in Supplementary Table S1. (**c**,**d**) Representative microphotographs of the significant increase in the Bcl-2-positive cells in the DG after GAL and Y1R agonist co-injection (**d**) compared with the control group (**c**). The cells in green are BDNF-positive using confocal laser microscopy. White arrows point to Bcl-2-IR cells. Dashed lines outline the Gcl of the dentate gyrus. The nuclei are shown in blue by DAPI. Abbreviations: aCSF = Control (artificial cerebrospinal fluid), GAL = galanin (3 nmol), Y1R agonist = Y1R receptor agonist [Leu^31^-Pro^34^]NPY (3 nmol), GAL + Y1R = co-administration of GAL and Y1R, GAL + Y1R + M871 = co-administration of GAL, Y1R, and GALR2 antagonist M871 (3 nmol).

Bcl-2-positive cells were explicitly found in the sgz of the dorsal DG (Figure 3a). Quantification of Bcl-2 cells lacked effects after GAL or the Y1R agonist alone compared to the control (Figure 3b). However, the co-injection of GAL and the YR1 agonist significantly increased (one-way ANOVA, F4, 15 = 4.76, *p* < 0.05) the number of Bcl-2-positive cells in the dorsal hippocampal DG (Figure 3b,d) compared to GAL, YR1 agonist, or aCSF groups (Newman–Keuls post-hoc test: *p* < 0.05) (Figure 3a–d). Similar to the BDNF-IR profile’s response described above, the presence of the GALR2 antagonist M871 completely blocked this increase (Newman–Keuls post-hoc test: *p* < 0.05) (Figure 3b), demonstrating the involvement of GALR2 in this interaction. Statistical values are presented in Supplementary Table S1.

### 3.3. GALR2 Agonist and Y1R Agonist Interaction Enhanced Survival and Neurite Outgrowth on Hippocampal Neuronal Cells

**Figure 4:**
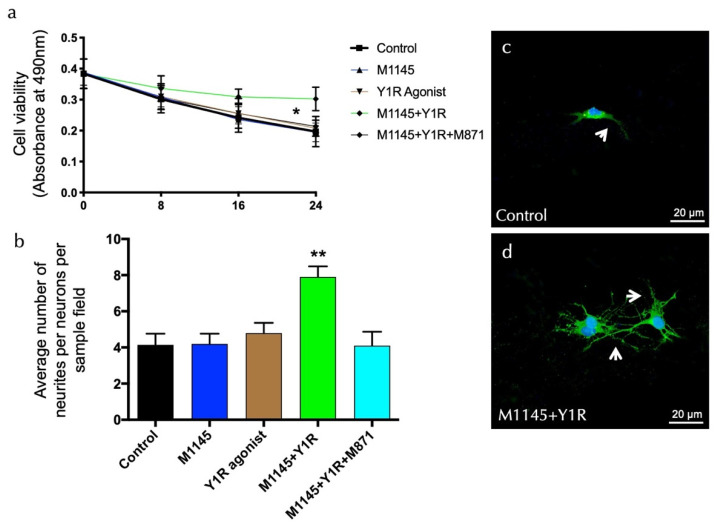
Evaluation of survival and neurite outgrowth on hippocampal neuronal cells. (**a**) Cell viability was determined by the MTS assay (CellTiter 96A Aqueous One Solution Cell Proliferation Assay (Promega)) with hippocampal neurons after treatment with the galanin receptor 2 agonist (M1145, 100 nM) and Y1R receptor agonist (100 nM), either alone or in combination with or without the GAL 2 receptor antagonist (M871, 1 µM). Cells (20,000 per well) were seeded, and after seven days, the cells were incubated for 0, 8, 16, and 24 h in triplicates with the different groups. Blanks (medium only plus CellTiter 96A Aqueous One Solution Reagent) were subtracted from the value measured for each well incubated with the groups. M1145 and Y1R agonists’ incubation significantly increased neuronal survival in the dorsal DG. This effect was blocked by treatment with the GALR2 antagonist M871. * *p* < 0.05, 24 h after M1145 and Y1R agonist co-stimulation compared with every group according to Student’s unpaired *t*-test statistical analysis. Statistical values are presented in Supplementary Table S1. (**b**–**d**) GALR2 and Y1R agonists’ modulation of neurites’ outgrowth. Primary hippocampal neurons were treated for 24 h without (control) or with M1145 (100 nM) and/or Y1R agonists (100 nM) with or without the GAL 2 receptor antagonist (M871, 1 µM). The numbers of neurites per cell were determined after immunofluorescent labeling of neurons and neuronal nuclei (Pan Neuronal Marker (ABN2300)/neuronal nuclei (DAPI)). Quantification is shown in Figure 4b, where the data are presented as mean ± SEM. The combined group is significantly different from the rest of the groups (** *p* < 0.01 vs. the rest of the groups according to one-way ANOVA followed by Newman–Keuls post-hoc test). Statistical values are presented in Supplementary Table S1. Representative microphotographs of the significant increase in the number of neurites in the hippocampal cells after M1145 and Y1R agonist treatment (**d**) compared with the control group (**c**). The cells in green are hippocampal neuron-positive using confocal laser microscopy. White arrows point to neurite extensions. The nuclei are counterstained in blue by DAPI. Abbreviations: Control = Culture medium, M1145 = galanin 2 receptor agonist (100 nM), Y1R agonist = Y1R receptor agonist [Leu^31^-Pro^34^]NPY (100 nM), M1145 + Y1R = co-administration of M1145 and Y1R, M1145 + Y1R + M871 = co-administration of M1145, Y1R, and GALR2 antagonist (1 µM).

To detect overall neuronal viability modulating effects, the effects of the GALR2 agonist M1145 and/or the Y1R agonist were monitored in an MTS cell proliferation assay after incubating cells with each substance in a time-course manner. The results of these experiments are summarized in Figure 4a. Upon incubation with M1145 and the Y1R agonist, hippocampal neurons’ viability was stable and resulted in an increased rate of survival at 24 h, as compared with the control (t = 2.535, df = 14; *p* < 0.05), M1145 (t = 2.765, df = 14; *p* < 0.05), and Y1R agonist (t = 2.649, df = 14; *p* < 0.05) groups (Figure 4a). Moreover, the specific GALR2 antagonist M871 abolished this effect (t = 2.755, df = 14; *p* < 0.05) (Figure 4b), demonstrating that this effect was mediated through the coactivation of GALR2 and NPYY1R. Statistical values are presented in Supplementary Table S1. However, incubation with M1145 or the Y1 agonist alone revealed a minor influence on neuronal viability compared to the control. These results indicate that the co-incubation of the GALR2 agonist and the Y1R agonist in solution exerts a profound effect on the survival of neurons.

In the second set of experiments, we determined morphologic details of the effects of M1145 and the Y1R agonist by analyzing neurite density in primary hippocampal cultures. The data show a significant synergistic increase of mean neurite number upon coactivation of M145 and the Y1R agonist for 24 h (one-way ANOVA, F4, 20 = 6.81, *p* < 0.01) compared to M1145, YR1 agonist, or control groups (Newman–Keuls post-hoc test: *p* < 0.01) (Figure 4b–d). The GALR2 antagonist M871 entirely blocks the synergic effects (Newman–Keuls post-hoc test: *p* < 0.01) (Figure 4b). Statistical values are presented in Supplementary Table S1.

### 3.4. Enhancement of Spatial Memory Consolidation after GAL and Y1R Agonist Co-Administration

**Figure 5:**
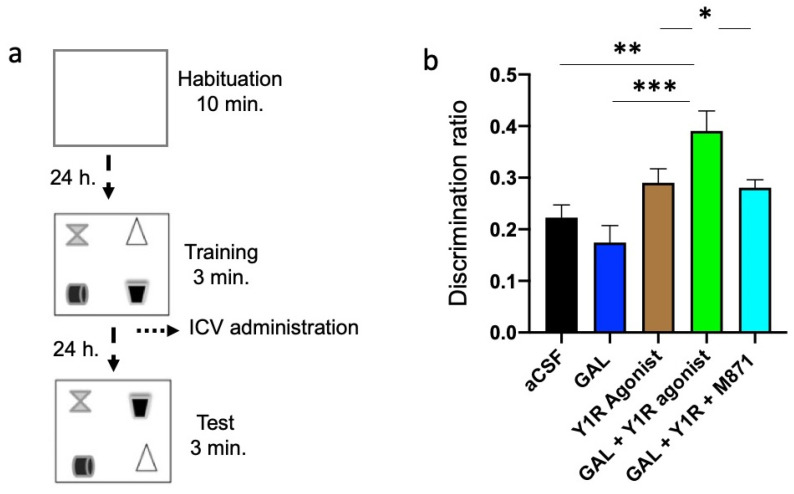
Spatial memory assessment after GAL and the Y1R agonist alone and combined in the object-in-place memory task. (**a**) Schematic representation of the trials completed in the object-in-place task. The animals performed the task in three phases, divided 24 h from each other, where they explored freely for ten minutes in the habituation phase without objects, three minutes in the training phase with four different objects, and finally, three minutes in the test phase with two of the objects with the exchanged position. To achieve memory consolidation, the pharmacological treatments were administered intracerebroventricularly (icv) to the different groups of animals 24 h before the testing phase. (**b**) Performance on the object-in-place task showing the ability of rats to discriminate the exchanged objects at 24 h post-training after the icv administration of GAL in combination with the Y1R agonist. An improvement in the object-in-place performance was observed after GAL and the Y1 agonist co-administration following a 24 h delay. Besides, this effect is counteracted by the GAL 2 receptor (GALR2) antagonist M871. Data are presented as the mean ± SEM of the discrimination ratio on the test phase. n = 6 animals in each group. * *p* < 0.05 vs. Y1R agonist and GAL + Y1R + M871; ** *p* < 0.01 vs. aCSF; *** *p* < 0.001 vs. GAL according to one-way ANOVA (F4, 25 = 3.56) followed by Newman–Keuls post-hoc test. Statistical values are presented in Supplementary Table S1. Abbreviations: aCSF = Control (artificial cerebrospinal fluid), GAL = galanin (3 nmol), Y1R agonist = Y1R receptor agonist [Leu^31^-Pro^34^]NPY (3 nmol), GAL + Y1R = co-administration of GAL and Y1R, GAL + Y1R + M871 = co-administration of GAL, Y1R, and GALR2 antagonist M871 (3 nmol).

We performed the object-in-place task to achieve the functional outcome related to the findings on the dorsal hippocampus after GAL and Y1R agonist co-administration. Rats explore freely for ten minutes during the habituation phase without objects and for three minutes in the training phase with four different objects. Twenty-four hours after the intracerebroventricular (icv) injections, animals were exposed to the test phase for three minutes with two objects with exchanged positions to assess drug effects on spatial memory learning (Figure 5a).

GAL and Y1R co-administration after the acquisition phase improved object-in-place memory consolidation after a 24 h period compared with aCSF (one-way ANOVA, F4, 25 = 7.98, *p* < 0.001; Newman–Keuls post-hoc test: *p* < 0.01; Figure 5b), GAL (Newman–Keuls post-hoc test: *p* < 0.001; Figure 5b), and Y1R agonist (Newman–Keuls post-hoc test: *p* < 0.05; Figure 5b) groups. GALR2 participation in this effect was achieved since the addition of the GALR2 antagonist M871 neutralized the enhanced memory performance (Newman–Keuls post-hoc test: *p* < 0.05; Figure 5b) induced by the co-administration of GAL and Y1R agonists in the object-in-place task. However, galanin (GAL) administration alone or the Y1R agonist alone lacked effects on the object-in-place memory task (Figure 5b) compared with the control group. Statistical values are presented in Supplementary Table S1.

Moreover, the total exploration time was analyzed during the training and test sessions. We observed that the exploration capacity of the animals was not affected by the treatments (Supplementary Table S2). Overall, the animals had a significant preference for the objects that were exchanged compared with objects that remained at the same location, as evidenced by within-group analyses: Control (t = 7.05; df = 5; *p* < 0.001), GAL (t = 5.77; df = 5; *p* < 0.001), Y1R agonist (t = 14.70; df = 5; *p* < 0.001), GAL + Y1R (t = 10.79; df = 5; *p* < 0.001), and GAL + Y1R + M871 (t = 14.89; df = 5; *p* < 0.001). Furthermore, the time exploring the relocated objects was significantly higher in the GAL + Y1R group compared with aCSF (one-way ANOVA, F4, 25 = 6.26, *p* < 0.01; Newman–Keuls post-hoc test: *p* < 0.01; Supplementary Table S2), GAL (Newman–Keuls post-hoc test: *p* < 0.001; Supplementary Table S2), Y1R agonist (Newman–Keuls post-hoc test: *p* < 0.05; Supplementary Table S2), and GAL + Y1R + M871 (Newman–Keuls post-hoc test: *p* < 0.05; Supplementary Table S2) groups. Spontaneous motor behavior showed no treatment effects (data of the locomotor activity is shown in Supplementary Table S3).

## 4. Discussion

We have demonstrated that GAL and Y1R agonist co-administration increased cell proliferation in the dorsal dentate gyrus (DG) of the hippocampus by using the proliferating cell nuclear antigen (PCNA). In agreement, we have recently observed the ability of the co-agonist treatment to enhance the cell proliferation in the DG of the ventral hippocampus throughout 5-Bromo-2′-deoxyuridine (BrdU) expression analysis at 24 h [32]. The Y1R agonist alone increased cell proliferation in the ventral DG in this previous work. However, we observed that the Y1R agonist lacks effects on cell proliferation in the dorsal DG. These findings suggest a differential role for NPY in subregions of the hippocampal formation, conferring functional differences between ventral and dorsal parts [1,3,42]. Accordingly, our results agree with previous evidence showing no differences in the dorsal hippocampus in the DG cell proliferation after NPY injection under physiological conditions in rats [22]. Further experiments using Y1R antagonists might uncover NPY’s tonic actions and its neurotrophic potential through Y1R.

In addition, species-specific differences between rats and mice in neurogenesis have been reported [43,44]. Thus, previous studies indicating that in physiological conditions exogenous NPY promotes DG cell proliferation on the dorsal hippocampus were performed on mice or in vitro [15,16]. Furthermore, we may speculate a different role exerted by NPY in pathological conditions, in which neurogenesis is affected since NPY induced cell proliferation on dorsal DG in the TMT-induced model of hippocampal neurodegeneration [22]. In this way, further research is required to study NPY and GAL interactions in pathological models of neurodegeneration. Regarding the injection of GAL alone, we observed no effects on dorsal hippocampal cell proliferation. Previously, it was reported that GalR2/3 mediated the proliferative and trophic effects of GAL [29], indicating in subsequent studies a role for GALR3 [45]. However, these experiments referred to in vitro conditions, exhibiting significant differences in systems in vivo.

The cellular mechanisms related to the observed effects on cell proliferation after GAL and NPYY1R agonist co-administration seem to be mediated by increased BDNF expression on the dorsal hippocampal DG. BDNF belongs to a family of neurotrophins that have a crucial role in increasing neurogenesis through changes in proliferation and cell survival [23]. Recent evidence showed that physical exercise protects the brain from Alzheimer’s disease (AD) memory impairment through increasing hippocampal neurogenesis in a necessary combination with BDNF [46]. Thus, agents that promote the close correlation between dentate neurogenesis and BDNF, as seen under the GAL and Y1R agonist combination, might be the key to preventing or curing AD. In this respect, our data are consistent with previous results on the BDNF-related neuroprotective effect of NPY in models of neurodegeneration [47,48]. As discussed above, we found neither effects on BDNF nor Bcl-2 expression after the injection of the Y1R agonist alone. In agreement, NPY administration lacked effects on BDNF or Bcl-2 expression on the dorsal hippocampus in physiological conditions [47,49].

Interestingly, GAL and Y1R agonist co-administration is accompanied by an enhanced expression of the anti-apoptotic protein Bcl-2 on the dorsal DG. Bcl-2 protects the mitochondrial membrane by forming heterodimers with proapoptotic proteins, thus preventing the release of cytochrome c [50]. Transgenic overexpression of Bcl-2 in neuronal cells results in increased hippocampal neurogenesis caused by reducing cell death of neuronal progenitor cells and increasing their survival [13,51]. NPY was shown to stimulate cell proliferation via activation of the mitogen-activated protein kinase (MAPK) pathway, leading to induction of members of the Bcl-2 family and subsequent regulation of neurotrophic factors, such as BDNF [52]. Accordingly, we observed that the GALR2–Y1R interaction could lead to increased integration in the intracellular signaling, as in the MAPK pathway [32]. Consequently, we can assume that GAL and Y1R agonists exert their neuroprotective actions by modulating neurotrophins and anti-apoptotic pathways.

These cellular effects induced by GALR2 and the Y1R agonist were achieved in hippocampal cells by studying survival in a B27-deprived medium. GALR2 and Y1R agonist co-incubation reversed the B27 deprivation-induced reduction in survival of hippocampal cells. Growth medium B27 promotes the growth and survival of embryonic hippocampal neurons. In a previous study, deprivation of B27 growth medium similarly caused reduced hippocampal cell viability, as observed in [53]. Based on our previous data, enhanced MAPK intracellular signaling may mediate the actions of GALR2 and Y1R in B27-deprived hippocampal cells, as discussed above. Moreover, GALR2 and Y1R agonist co-incubation promoted the neurites’ outgrowth at 24 h in hippocampal cells, where BDNF might be a common mechanism in our in vivo and in vitro experiments. In agreement, it was shown that BDNF exerted a promoting effect on hippocampal survival and dendritic outgrowth in primary hippocampal cultures with B27 deprivation [54].

The functional outcome was validated by demonstrating the enhancement of spatial learning after GAL and Y1R agonist co-administration on the object-in-place task at 24 h. In humans, object-in-place memory is critical for everyday living, allowing the detection of a last event within an environment during a single encounter. The hippocampus has a manifest function in object recognition memory tasks with a spatial component, such as the object-in-place task [38,55,56]. Moreover, patients with neurodegenerative diseases, such as Alzheimer’s disease (AD), presented disturbed object-in-place associative recognition memory [57]. Subsequently, electrophysiological data proved that the spatial location of objects is detected by hippocampal neurons [58], and their lesion impairs object-in-place tasks [59,60]. Within the hippocampus, the selective inactivation of the DG subregion was shown to impair the encoding of object location memory [61]. Remarkably, the DG was the only hippocampal subregion found to decrease NPY-IR fibers in a rat model of AD [62]. In agreement with our results, genetically increased neurogenesis on the dorsal DG of the hippocampus improved spatial learning in aging rodents [63]. However, previous evidence has shown that proliferating dentate granule cells achieve functional integration into the hippocampal neuronal network as early as two weeks after birth [64,65]. We may speculate that the molecular mechanisms underlying the memory-enhancing effects of the Y1R agonist and GAL at 24 h could be mediated by enhancing the signaling of these two protomers in the Y1R–GALR2 heterocomplexes in the neurogenic zone of the dorsal hippocampus. Moreover, our in vivo and vitro findings, regarding a BDNF enhancement in the dorsal dentate gyrus and on survival and neurites’ outgrowth on hippocampal neurons after 24 h, might also support the contribution of BDNF in this mechanism. Interestingly, running was shown to enhance neuronal proliferation and BDNF signaling on the dorsal hippocampus related with augmented pattern separation during memory tasks at a 24 h delay after training in rodents [66] and humans [67]. Thus, the increased signaling for both Y1R–GALR2 heterocomplexes and BDNF could induce plasticity in the encoded hippocampal networks to enhance the connections that will be reactivated during retrieval 24 h later. Accordingly, immature granule cells of the hippocampus have been shown to be more excitable and to have enhanced plasticity compared to mature neurons [68,69]. Besides, the neuroprotective molecule P7C3 increased neuronal cell proliferation and the BDNF signaling in the dorsal hippocampus associated with improved spatial memory learning effects at 24 h in neurodegenerative rodent models [70,71]. In the formation of the long-term spatial memory, it is proposed that it may be brought through the increased formation of BDNF–TrkB signaling complexes and the anti-apoptotic factor Bcl-2 in the hippocampal nerve cells, enriched in activated Y1R-GALR2. Through activation of TrkB and Bcl-2 complexes, transcription factors can be modulated to produce adaptor proteins that bind to and stabilize the short-term memory into a long-term memory through combined binding of the adaptor proteins to two or more heteroreceptor complexes, and combined binding of the cytoskeleton/postsynaptic proteins and the hetero/homo-receptor complexes [72,73]. To study the long-term effects of the Y1R agonist and GAL on spatial memory, related to the survival and integration of proliferating cells in the dorsal hippocampal circuit, further research is required.

## 5. Conclusions

Taken together, the Y1R agonist and GAL may promote cell proliferation in the DG of the dorsal hippocampus and the induction of neuroprotective factors, such as BDNF and Bcl-2. These effects may be mediated by Y1R–GALR2 heteroreceptor complexes [25,26,27,32] to mediate increased survival and neurites’ outgrowth observed on neuronal hippocampal cells. Accordingly, these cellular effects may be linked to the improved spatial memory effects observed. In this way, the short-term memory of the object-in-place task can be achieved through a reorganization of the signaling in this Y1R–GALR2 heteroreceptor complex, including the homo-receptor complex associated with altered hetero/homo-signaling that represents the augmented spatial memory. Our data may suggest the development of heterobivalent agonist pharmacophores, targeting Y1R–GALR2 heterocomplexes, therefore acting on the neuronal precursor cells of the DG in the dorsal hippocampus for the novel therapy of neurodegenerative cognitive-affecting diseases.

## 6. Patents

Patent P202030533 with WO 2021/245315 partially resulted from the work reported in this manuscript.

## Data Availability

The original contributions presented in this work are included in the article and Supplementary Material, further inquiries can be directed to the corresponding authors.

## Supplementary Materials

The following supporting information can be downloaded at: https://www.mdpi.com/article/10.3390/biomedicines10061297/s1, Table S1: Statistical values presented in tabular form for Figure 1, Figure 2, Figure 3, Figure 4 and Figure 5. Table S2: Exploratory activity of rats treated with galanin (GAL) and the neuropeptide Y (NPY) Y1 receptor agonist (Y1R agonist) alone and in combination. Table S3: Locomotor/spontaneous activity in the object-in-place task.

## References

[1] Baptista P., Andrade J.P. (2018). Adult Hippocampal Neurogenesis: Regulation and Possible Functional and Clinical Correlates. Front. Neuroanat..

[2] Spalding K.L., Bergmann O., Alkass K., Bernard S., Salehpour M., Huttner H.B., Bostrom E., Westerlund I., Vial C., Buchholz B.A. (2013). Dynamics of hippocampal neurogenesis in adult humans. Cell.

[3] Fanselow M.S., Dong H.W. (2010). Are the dorsal and ventral hippocampus functionally distinct structures?. Neuron.

[4] Kheirbek M.A., Drew L.J., Burghardt N.S., Costantini D.O., Tannenholz L., Ahmari S.E., Zeng H., Fenton A.A., Hen R. (2013). Differential control of learning and anxiety along the dorsoventral axis of the dentate gyrus. Neuron.

[5] Tanti A., Belzung C. (2013). Neurogenesis along the septo-temporal axis of the hippocampus: Are depression and the action of antidepressants region-specific?. Neuroscience.

[6] Moreno-Jimenez E.P., Flor-Garcia M., Terreros-Roncal J., Rabano A., Cafini F., Pallas-Bazarra N., Avila J., Llorens-Martin M. (2019). Adult hippocampal neurogenesis is abundant in neurologically healthy subjects and drops sharply in patients with Alzheimer’s disease. Nat. Med..

[7] Boldrini M., Fulmore C.A., Tartt A.N., Simeon L.R., Pavlova I., Poposka V., Rosoklija G.B., Stankov A., Arango V., Dwork A.J. (2018). Human Hippocampal Neurogenesis Persists throughout Aging. Cell Stem Cell.

[8] Terreros-Roncal J., Moreno-Jimenez E.P., Flor-Garcia M., Rodriguez-Moreno C.B., Trinchero M.F., Marquez-Valadez B., Cafini F., Rabano A., Llorens-Martin M. (2022). Response to Comment on “Impact of neurodegenerative diseases on human adult hippocampal neurogenesis”. Science.

[9] Kim I.B., Park S.C. (2021). Neural Circuitry-Neurogenesis Coupling Model of Depression. Int. J. Mol. Sci..

[10] Martos D., Tuka B., Tanaka M., Vecsei L., Telegdy G. (2022). Memory Enhancement with Kynurenic Acid and Its Mechanisms in Neurotransmission. Biomedicines.

[11] Colucci-D’Amato L., Speranza L., Volpicelli F. (2020). Neurotrophic Factor BDNF, Physiological Functions and Therapeutic Potential in Depression, Neurodegeneration and Brain Cancer. Int. J. Mol. Sci..

[12] Kempermann G., Song H., Gage F.H. (2015). Neurogenesis in the Adult Hippocampus. Cold Spring Harb. Perspect. Biol..

[13] Kuhn H.G. (2015). Control of Cell Survival in Adult Mammalian Neurogenesis. Cold Spring Harb. Perspect. Biol..

[14] Zaben M.J., Gray W.P. (2013). Neuropeptides and hippocampal neurogenesis. Neuropeptides.

[15] Decressac M., Wright B., David B., Tyers P., Jaber M., Barker R.A., Gaillard A. (2011). Exogenous neuropeptide Y promotes in vivo hippocampal neurogenesis. Hippocampus.

[16] Howell O.W., Silva S., Scharfman H.E., Sosunov A.A., Zaben M., Shtaya A., McKhann G., Herzog H., Laskowski A., Gray W.P. (2007). Neuropeptide Y is important for basal and seizure-induced precursor cell proliferation in the hippocampus. Neurobiol. Dis..

[17] Hadad-Ophir O., Albrecht A., Stork O., Richter-Levin G. (2014). Amygdala activation and GABAergic gene expression in hippocampal sub-regions at the interplay of stress and spatial learning. Front. Behav. Neurosci..

[18] Borbely E., Scheich B., Helyes Z. (2013). Neuropeptides in learning and memory. Neuropeptides.

[19] Martel J.C., Alagar R., Robitaille Y., Quirion R. (1990). Neuropeptide Y receptor binding sites in human brain. Possible alteration in Alzheimer’s disease. Brain Res..

[20] Nilsson C.L., Brinkmalm A., Minthon L., Blennow K., Ekman R. (2001). Processing of neuropeptide Y, galanin, and somatostatin in the cerebrospinal fluid of patients with Alzheimer’s disease and frontotemporal dementia. Peptides.

[21] Gotzsche C.R., Woldbye D.P. (2016). The role of NPY in learning and memory. Neuropeptides.

[22] Corvino V., Marchese E., Podda M.V., Lattanzi W., Giannetti S., Di Maria V., Cocco S., Grassi C., Michetti F., Geloso M.C. (2014). The neurogenic effects of exogenous neuropeptide Y: Early molecular events and long-lasting effects in the hippocampus of trimethyltin-treated rats. PLoS ONE.

[23] Miranda M., Morici J.F., Zanoni M.B., Bekinschtein P. (2019). Brain-Derived Neurotrophic Factor: A Key Molecule for Memory in the Healthy and the Pathological Brain. Front. Cell. Neurosci..

[24] Abuaish S., Al-Otaibi N.M., Abujamel T.S., Alzahrani S.A., Alotaibi S.M., AlShawakir Y.A., Aabed K., El-Ansary A. (2021). Fecal Transplant and Bifidobacterium Treatments Modulate Gut Clostridium Bacteria and Rescue Social Impairment and Hippocampal BDNF Expression in a Rodent Model of Autism. Brain Sci..

[25] Narvaez M., Millon C., Borroto-Escuela D., Flores-Burgess A., Santin L., Parrado C., Gago B., Puigcerver A., Fuxe K., Narvaez J.A. (2015). Galanin receptor 2-neuropeptide Y Y1 receptor interactions in the amygdala lead to increased anxiolytic actions. Brain Struct. Funct..

[26] Narvaez M., Borroto-Escuela D.O., Millon C., Gago B., Flores-Burgess A., Santin L., Fuxe K., Narvaez J.A., Diaz-Cabiale Z. (2016). Galanin receptor 2-neuropeptide Y Y1 receptor interactions in the dentate gyrus are related with antidepressant-like effects. Brain Struct. Funct..

[27] Narvaez M., Borroto-Escuela D.O., Santin L., Millon C., Gago B., Flores-Burgess A., Barbancho M.A., Perez de la Mora M., Narvaez J., Diaz-Cabiale Z. (2018). A Novel Integrative Mechanism in Anxiolytic Behavior Induced by Galanin 2/Neuropeptide Y Y1 Receptor Interactions on Medial Paracapsular Intercalated Amygdala in Rats. Front. Cell. Neurosci..

[28] Katsetos C.D., Del Valle L., Geddes J.F., Assimakopoulou M., Legido A., Boyd J.C., Balin B., Parikh N.A., Maraziotis T., de Chadarevian J.P. (2001). Aberrant localization of the neuronal class III beta-tubulin in astrocytomas. Arch. Pathol. Lab. Med..

[29] Abbosh C., Lawkowski A., Zaben M., Gray W. (2011). GalR2/3 mediates proliferative and trophic effects of galanin on postnatal hippocampal precursors. J. Neurochem..

[30] Beck B., Pourie G. (2013). Ghrelin, neuropeptide Y, and other feeding-regulatory peptides active in the hippocampus: Role in learning and memory. Nutr. Rev..

[31] Li L., Yu L., Kong Q. (2013). Exogenous galanin attenuates spatial memory impairment and decreases hippocampal beta-amyloid levels in rat model of Alzheimer’s disease. Int. J. Neurosci..

[32] Borroto-Escuela D.O., Pita-Rodriguez M., Fores-Pons R., Barbancho M.A., Fuxe K., Narvaez M. (2021). Galanin and neuropeptide Y interactions elicit antidepressant activity linked to neuronal precursor cells of the dentate gyrus in the ventral hippocampus. J. Cell. Physiol..

[33] Paxinos G., Watson C. (2006). The Rat Brain in Stereotaxic Coordinates: Hard Cover Edition.

[34] Gundersen H.J., Bagger P., Bendtsen T.F., Evans S.M., Korbo L., Marcussen N., Moller A., Nielsen K., Nyengaard J.R., Pakkenberg B. (1988). The new stereological tools: Disector, fractionator, nucleator and point sampled intercepts and their use in pathological research and diagnosis. APMIS.

[35] Cohen H., Zohar J., Kaplan Z., Arnt J. (2018). Adjunctive treatment with brexpiprazole and escitalopram reduces behavioral stress responses and increase hypothalamic NPY immunoreactivity in a rat model of PTSD-like symptoms. Eur. Neuropsychopharmacol..

[36] Rapp A., Brandl N., Volpi N., Huettinger M. (2005). Evaluation of chondroitin sulfate bioactivity in hippocampal neurones and the astrocyte cell line U373: Influence of position of sulfate groups and charge density. Basic Clin. Pharmacol. Toxicol..

[37] Narvaez M., Andrade-Talavera Y., Valladolid-Acebes I., Fredriksson M., Siegele P., Hernandez-Sosa A., Fisahn A., Fuxe K., Borroto-Escuela D.O. (2020). Existence of FGFR1-5-HT1AR heteroreceptor complexes in hippocampal astrocytes. Putative link to 5-HT and FGF2 modulation of hippocampal gamma oscillations. Neuropharmacology.

[38] Warburton E.C., Brown M.W. (2015). Neural circuitry for rat recognition memory. Behav. Brain Res..

[39] Harrison F.E., Hosseini A.H., McDonald M.P. (2009). Endogenous anxiety and stress responses in water maze and Barnes maze spatial memory tasks. Behav. Brain Res..

[40] Ampuero E., Stehberg J., Gonzalez D., Besser N., Ferrero M., Diaz-Veliz G., Wyneken U., Rubio F.J. (2013). Repetitive fluoxetine treatment affects long-term memories but not learning. Behav. Brain Res..

[41] Barker G.R., Warburton E.C. (2015). Object-in-place associative recognition memory depends on glutamate receptor neurotransmission within two defined hippocampal-cortical circuits: A critical role for AMPA and NMDA receptors in the hippocampus, perirhinal, and prefrontal cortices. Cereb. Cortex.

[42] Lee A.R., Kim J.H., Cho E., Kim M., Park M. (2017). Dorsal and Ventral Hippocampus Differentiate in Functional Pathways and Differentially Associate with Neurological Disease-Related Genes during Postnatal Development. Front. Mol. Neurosci..

[43] Ray J., Gage F.H. (2006). Differential properties of adult rat and mouse brain-derived neural stem/progenitor cells. Mol. Cell. Neurosci..

[44] Radic T., Friess L., Vijikumar A., Jungenitz T., Deller T., Schwarzacher S.W. (2017). Differential Postnatal Expression of Neuronal Maturation Markers in the Dentate Gyrus of Mice and Rats. Front. Neuroanat..

[45] Khan D., Khan M., Runesson J., Zaben M., Gray W.P. (2017). GalR3 mediates galanin proliferative effects on postnatal hippocampal precursors. Neuropeptides.

[46] Choi S.H., Bylykbashi E., Chatila Z.K., Lee S.W., Pulli B., Clemenson G.D., Kim E., Rompala A., Oram M.K., Asselin C. (2018). Combined adult neurogenesis and BDNF mimic exercise effects on cognition in an Alzheimer’s mouse model. Science.

[47] Corvino V., Marchese E., Giannetti S., Lattanzi W., Bonvissuto D., Biamonte F., Mongiovi A.M., Michetti F., Geloso M.C. (2012). The neuroprotective and neurogenic effects of neuropeptide Y administration in an animal model of hippocampal neurodegeneration and temporal lobe epilepsy induced by trimethyltin. J. Neurochem..

[48] Croce N., Dinallo V., Ricci V., Federici G., Caltagirone C., Bernardini S., Angelucci F. (2011). Neuroprotective effect of neuropeptide Y against beta-amyloid 25–35 toxicity in SH-SY5Y neuroblastoma cells is associated with increased neurotrophin production. Neurodegener. Dis..

[49] Cohen H., Liu T., Kozlovsky N., Kaplan Z., Zohar J., Mathe A.A. (2012). The neuropeptide Y (NPY)-ergic system is associated with behavioral resilience to stress exposure in an animal model of post-traumatic stress disorder. Neuropsychopharmacology.

[50] Renault T.T., Teijido O., Antonsson B., Dejean L.M., Manon S. (2013). Regulation of Bax mitochondrial localization by Bcl-2 and Bcl-x(L): Keep your friends close but your enemies closer. Int. J. Biochem. Cell. Biol..

[51] Sasaki T., Kitagawa K., Yagita Y., Sugiura S., Omura-Matsuoka E., Tanaka S., Matsushita K., Okano H., Tsujimoto Y., Hori M. (2006). Bcl2 enhances survival of newborn neurons in the normal and ischemic hippocampus. J. Neurosci. Res..

[52] Li Z., Zhang J., Liu Z., Woo C.W., Thiele C.J. (2007). Downregulation of Bim by brain-derived neurotrophic factor activation of TrkB protects neuroblastoma cells from paclitaxel but not etoposide or cisplatin-induced cell death. Cell Death Differ..

[53] Sunwoldt J., Bosche B., Meisel A., Mergenthaler P. (2017). Neuronal Culture Microenvironments Determine Preferences in Bioenergetic Pathway Use. Front. Mol. Neurosci..

[54] Park S.W., Nhu L.H., Cho H.Y., Seo M.K., Lee C.H., Ly N.N., Choi C.M., Lee B.J., Kim G.M., Seol W. (2016). p11 mediates the BDNF-protective effects in dendritic outgrowth and spine formation in B27-deprived primary hippocampal cells. J. Affect. Disord..

[55] Owen A.M., Sahakian B.J., Semple J., Polkey C.E., Robbins T.W. (1995). Visuo-spatial short-term recognition memory and learning after temporal lobe excisions, frontal lobe excisions or amygdalo-hippocampectomy in man. Neuropsychologia.

[56] Milner B., Johnsrude I., Crane J. (1997). Right medial temporal-lobe contribution to object-location memory. Philos. Trans. R. Soc. Lond. Ser. B Biol. Sci..

[57] Fowler K.S., Saling M.M., Conway E.L., Semple J.M., Louis W.J. (2002). Paired associate performance in the early detection of DAT. J. Int. Neuropsychol. Soc..

[58] Muller R. (1996). A quarter of a century of place cells. Neuron.

[59] Barker G.R., Warburton E.C. (2011). When is the hippocampus involved in recognition memory?. J. Neurosci..

[60] Mumby D.G., Gaskin S., Glenn M.J., Schramek T.E., Lehmann H. (2002). Hippocampal damage and exploratory preferences in rats: Memory for objects, places, and contexts. Learn. Mem..

[61] Barbosa F.F., Pontes I.M., Ribeiro S., Ribeiro A.M., Silva R.H. (2012). Differential roles of the dorsal hippocampal regions in the acquisition of spatial and temporal aspects of episodic-like memory. Behav. Brain Res..

[62] Rangani R.J., Upadhya M.A., Nakhate K.T., Kokare D.M., Subhedar N.K. (2012). Nicotine evoked improvement in learning and memory is mediated through NPY Y1 receptors in rat model of Alzheimer’s disease. Peptides.

[63] Berdugo-Vega G., Arias-Gil G., Lopez-Fernandez A., Artegiani B., Wasielewska J.M., Lee C.C., Lippert M.T., Kempermann G., Takagaki K., Calegari F. (2020). Increasing neurogenesis refines hippocampal activity rejuvenating navigational learning strategies and contextual memory throughout life. Nat. Commun..

[64] Nakashiba T., Cushman J.D., Pelkey K.A., Renaudineau S., Buhl D.L., McHugh T.J., Rodriguez Barrera V., Chittajallu R., Iwamoto K.S., McBain C.J. (2012). Young dentate granule cells mediate pattern separation, whereas old granule cells facilitate pattern completion. Cell.

[65] Gu Y., Arruda-Carvalho M., Wang J., Janoschka S.R., Josselyn S.A., Frankland P.W., Ge S. (2012). Optical controlling reveals time-dependent roles for adult-born dentate granule cells. Nat. Neurosci..

[66] Bolz L., Heigele S., Bischofberger J. (2015). Running Improves Pattern Separation during Novel Object Recognition. Brain Plast..

[67] Butavand D.R., Rodríguez M.F., Cifuentes M.V., Miranda M., Bauza C.G., Bekinschtein P., Ballarini F. (2021). Acute and chronic physical activity improve spatial pattern separation in humans. bioRxiv.

[68] Ge S., Yang C.H., Hsu K.S., Ming G.L., Song H. (2007). A critical period for enhanced synaptic plasticity in newly generated neurons of the adult brain. Neuron.

[69] Schmidt-Hieber C., Jonas P., Bischofberger J. (2004). Enhanced synaptic plasticity in newly generated granule cells of the adult hippocampus. Nature.

[70] Loris Z.B., Pieper A.A., Dietrich W.D. (2017). The neuroprotective compound P7C3-A20 promotes neurogenesis and improves cognitive function after ischemic stroke. Exp. Neurol..

[71] Jiang B., Song L., Huang C., Zhang W. (2016). P7C3 Attenuates the Scopolamine-Induced Memory Impairments in C57BL/6J Mice. Neurochem. Res..

[72] Fuxe K., Agnati L.F., Borroto-Escuela D.O. (2014). The impact of receptor-receptor interactions in heteroreceptor complexes on brain plasticity. Expert Rev. Neurother..

[73] Borroto-Escuela D.O., Agnati L.F., Bechter K., Jansson A., Tarakanov A.O., Fuxe K. (2015). The role of transmitter diffusion and flow versus extracellular vesicles in volume transmission in the brain neural-glial networks. Philos. Trans. R. Soc. Lond. Ser. B Biol. Sci..

