# Supplementary material for: Galanin and Neuropeptide Y Interaction Enhances Proliferation of Granule Precursor Cells and Expression of Neuroprotective Factors in the Rat Hippocampus with Consequent Augmented Spatial Memory
Source: Biomedicines. 2022 Jun 1;10(6):1297. doi: 10.3390/biomedicines10061297 (PMC9219743; doi:10.3390/biomedicines10061297)
Supplement: Supplementary file 1 [file biomedicines-10-01297-s001.zip › biomedicines-1738592-supplementary.pdf]

Supplementary Material

Galanin and Neuropeptide Y interaction enhances proliferation of granule precursor cells and expression of neuroprotective factors in the rat hippocampus with consequent augmented spatial memory

Marina Mirchandani-Duque +1; Miguel A. Barbancho +1; Alexander López-Salas 1; Jose Erik Alvarez-Contino 1; Natalia García-Casares 1; Kjell Fuxe 2; Dasiel O. Borroto-Escuela *1,2,3 and Manuel Narváez*1,2.

1 Instituto de Investigación Biomédica de Málaga, Facultad de Medicina, Universidad de Málaga, Málaga, Spain,

2 Department of Neuroscience, Karolinska Institute, Stockholm, Sweden,

3 Department of Biomolecular Science, Section of Physiology, University of Urbino, Urbino, Italy,

+ These authors contribute equally to this work.

*Correspondence:

Manuel Narváez, mnarvaez@uma.es; Dasiel Borroto escuela: dasiel@uma.es

Intracerebral cannulations

Rats anesthetized intraperitoneally with Equitesin (3,3ml/Kg; chloral hydrate 2.1 g, sodium pentobarbital 0.46 g, MgSO4 1.06 g, propylene glycol 21.4 ml, ethanol (90%) 5.7 ml, H_2O 3 ml; Sigma Aldrich) were implanted with a chronic 22-gauge stainless-steel guide cannula (Plastics One In) into the right lateral cerebral ventricle using the following stereotaxic coordinates: +1.4mm lateral, -1mm posterior to the bregma, and 3.6mm below the surface of the skull (Paxinos and Watson, 2006). After surgery, animals were individually housed and allowed recovery for 7 days. This method of anesthesia, cannulation and postsurgical

care has been previously standarized (Narváez et al., 2015; Narváez et al., 2016; Narváez et al., 2018; Borroto-Escuela et al., 2021).

Intracerebroventricular administration of peptides

Cannulated rats were randomly allocated to different groups. Peptides were freshly prepared, dissolved in aCSF and injected into the right lateral ventricle. The total volume was 5 µl per injection with an infusion time of 1 min. The NPYY1R agonist [Leu31,Pro34]NPY (Ki=0.39 nM for NPYY1R) and GALR2 antagonist M871 (Ki=13.1 and 420 nM for GALR2 and GALR1 respectively) were obtained from Tocris Bioscience (Bristol, UK). Experimental groups and the n size of each group is indicated in the different procedures. After the experiments, brains were removed, testing the placement of the cannula for icv injection by cutting the brain in the coronal plane in a Cryostat (HM550, Microm International). These procedures of intracerebroventricular (icv) injections and preparation of artificial cerebrospinal fluid (aCSF) have already been standarized in our laboratory (Narváez et al., 2015; Narváez et al., 2016; Narváez et al., 2018; Borroto-Escuela et al., 2021).

Counting Procedure

PCNA-labeled cells number was counted with an Olympus BX51 microscope, Olympus, Denmark interfaced with a computer and a colour JVC digital video camera. For stereological analysis, sampling of PCNA positive cells was performed throughout the dentate gyrus of the dorsal hippocampus in the rostrocaudal dimension using the optical fractionator. This method combines the optical dissector with a fractionator sampling scheme to exclude volume divergences (Gundersen et al., 1988). Counterstaining with phase contrast allowed delineation of different areas in each section (Paxinos and Watson, 2006). Numbers of PCNA positive cells were quantified in at least six representative 150 µm, evenly spaced sections per animal (4 rats per group). A random set of sampling frames with a known area (α frame) was generated for each section using the C.A.S.T. Grid (Olympus; Albertslund, Denmark). After the objects were counted (ΣQ-) the total number of positive cells were estimated as: N = ΣQ- x fs x fa x fh (Gundersen et al., 1988), where fs is the numerical fraction of the section used, fa is the areal fraction and fh is the linear fraction of section thickness. The quantification was limited to the granular cell layer and

subgranular zone. Subgranular region was outlined as a band-limited by three nuclei down from the edge between the granular cell layer and the hilar region, and cells located more than two cells away from the subgranular zone were excluded. The coefficient of error (CE) for each estimation and animal ranged from 0.05 to 0.1. The total CE of each group ranged from 0.07 to 0.08. Counting of labelled cells was set starting at 5 μm below the surface and focusing through the 20 μm section optical plane, and the number of counting frames used was 90-110 per animal. We have used this stereological procedure is previous studies (Narvaez et al., 2016;Narvaez et al., 2018).

Hippocampal Cell Culture and conditions

Rat primary hippocampal neuronal cells were purchased from QBM Cell Science (Montreal, Canada) and cultured in Neuro basal medium supplemented with 10% FBS, 2 mM GlutaMAX-1, 1 mM sodium pyruvate, 100 U/ml penicillin G, and 100 μg/ml streptomycin and 2% B-27 supplement (components: biotin, α-tocopherol acetate, α-tocopherol, vitamin A, bovine serum albumin [BSA], catalase, insulin, transferrin, superoxide dismutase, corticosterone, galactose, ethanolamine, glutathione, carnitine, linoleic acid, progesterone, putrescine, selenium, and triodo-I-thyronine) at 37°C in a humidified 10% CO2 environment according to manufacturer’s instructions. Half part of the medium was changed every 3 days. The cells were grown under the above conditions (control condition) for 7 d. Primary hippocampal nerve cells, starved for 3 h, were treated with the substances and time-course described in a B27-deprived medium for Hippocampal cells viability analysis.

For the analysis of neurite outgrowth a different set of cultured hippocampal neurons was grown in control conditions and then incubated with the indicated treatments for 24 hours. Cells were grown on poly-D-lysine-coated glass coverslips and fixed with 4% formaldehyde solution for 20 min followed by two washes with PBS containing 20 mM glycine to quench the aldehyde groups. Then, after permeabilization with PBS containing 0.2% Triton X-100 for 5 min, cells were treated with PBS containing 1% bovine serum albumin. After 1 h at room temperature, cells were labelled with Neuro-Chrom Pan Neuronal Marker primary antibody (ABN2300, 1:100, Sigma-Aldrich; Merck Life Science S.L.U.)

for 1 h, extensively washed, and stained with the green fluorescence secondary antibody goat anti-rabbit DyLight 488 (Jackson Laboratories InmunoResearch, 1:100). Samples were rinsed and visualized on a Leica SP2 confocal microscope.

Supplementary Table S1

Group	aCSF	GAL	Y1R agonist	GAL+Y1R	GAL+Y1R+M871
Estimated number of PCNA+ cells	660±35	660±26	700±35	870±51	670±25
Estimated number of BDNF+ cells	104±10	112±15	129±6	169±7	138±8
Estimated number of Bcl-2+ cells	77±5	79±8	87±3	113±8	92±9
Cell viability	0.2±0.033	0.19±0.03 (M1145)	0.21±0.025	0.3±0.024	0.21±0.02
Average number of neurites per neuron per sample field	4.1±0.62	4.2±0.57 (M1145)	4.8±0.57	7.9±0.58	4.1±0.76
Discrimination Ratio	0.22±0.02	0.17±0.03	0.29±0.03	0.39±0.04	0.28±0.02

Supplementary Table S1. Statistical values presented in tabular form for Figures 1, 2, 3, 4 and 5. Data are expressed as mean ± SEM. Statistical analysis are detailed in the manuscript and the figure legends.

Supplementary Table S2

Group	aCSF	GAL	Y1R agonist	GAL+Y1R	GAL+Y1R+M871
Training (Total exploration time (s))	20±0.6	20.8±0.3	20.5±0.7	20.9±0.7	21.1±1.2
Test (Total exploration time (s))	19.8±1.3	18.4±1.1	20.5±1.1	22.8±1	19.7±1.2
Familiar objects (s)	7.7±0.5	7.6±0.7	7.3±0.6	6.9±0.5	7.1±0.5
Relocated objects (s)	12.1±0.9***	10.7±0.5 ***&&&	13.1±0.5*** &	15.9±0.9*** &&	12.6±0.8*** &

Supplementary Table S2. Exploratory activity of rats treated with Galanin (GAL) and the Neuropeptide Y Y1 receptor agonist (Y1R agonist) alone and in combination. Data are expressed as mean ± SEM from the training and test sessions during the Object-in-place task. For total exploration time in training and

test sessions no statistically significant differences were observed between the experimental groups according to one way ANOVA. &p < 0.05 GAL+Y1R vs Y1R agonist and GAL+Y1R+M871; &&p < 0.01 GAL+Y1R vs aCSF; ; &&&p < 0.001 GAL+Y1R vs GAL according to one-way ANOVA followed by Newman-Keuls post-hoc test for relocated objects time. ***p < 0.001 (two-tailed paired t-test) Familiar vs. Relocated. Abbreviations: aCSF= control (artificial Cerebrospinal fluid); GAL = Galanin 3nmol; Y1R agonist = Y1R receptor agonist [Leu^{31}-Pro^{34}]NPY 3nmol; GAL+ Y1R = Coadministration of GAL and Y1R; GAL+ Y1R +M871 = Co-administration of M1145, Y1R and GALR2 antagonist M871 3nmol.

Supplementary Table S3

Group	aCSF	GAL	Y1R agonist	GAL+Y1R	GAL+Y1R+M871
Locomotor Activity (cm)	2070±116	2233±105	2081±103	2221±150	2111±90

Supplementary Table S3. Locomotor/spontaneous activity in the Object-in-place task. Data are expressed as mean ± SEM. According to one-way ANOVA, no statistically significant difference was observed between the experimental groups. Abbreviations: aCSF= control (artificial Cerebrospinal fluid); GAL = Galanin 3nmol; Y1R agonist = Y1R receptor agonist [Leu^{31}-Pro^{34}]NPY 3nmol; GAL+ Y1R = Coadministration of GAL and Y1R; GAL+ Y1R +M871 = Co-administration of M1145, Y1R and GALR2 antagonist M871 3nmol.

References

Borroto-Escuela, D.O., Pita-Rodriguez, M., Fores-Pons, R., Barbancho, M.A., Fuxe, K., and Narvaez, M. (2021). Galanin and neuropeptide Y interactions elicit antidepressant activity linked to neuronal precursor cells of the dentate gyrus in the ventral hippocampus. *J Cell Physiol* 236**,** 3565-3578.

Gundersen, H.J., Bagger, P., Bendtsen, T.F., Evans, S.M., Korbo, L., Marcussen, N., Moller, A., Nielsen, K., Nyengaard, J.R., Pakkenberg, B., and Et Al. (1988). The new stereological tools: disector, fractionator, nucleator and point sampled intercepts and their use in pathological research and diagnosis. APMIS 96, 857-881.

Narvaez, M., et al. (2015). "Galanin receptor 2-neuropeptide Y Y1 receptor interactions in the amygdala lead to increased anxiolytic actions." Brain Struct Funct 220(4): 2289-2301.

Narvaez, M., et al. (2016). "Galanin receptor 2-neuropeptide Y Y1 receptor interactions in the dentate gyrus are related with antidepressant-like effects." Brain Struct Funct 221(8): 4129-4139

Narvaez, M., et al. (2018). "A Novel Integrative Mechanism in Anxiolytic Behavior Induced by Galanin 2/Neuropeptide Y Y1 Receptor Interactions on Medial Paracapsular Intercalated Amygdala in Rats." Front Cell Neurosci 12: 119

Paxinos, G., and Watson, C. (2006). *The rat brain in stereotaxic coordinates: hard cover edition.* Elsevier.
